# Mapping Patient Pathways in Tuberculosis Care: Insights From Gujarat and Jharkhand States of India

**DOI:** 10.7759/cureus.67716

**Published:** 2024-08-25

**Authors:** Kunwar Pranav Deep, Richa Gwalani, Divya Singh, Seamone Baliyan, Juhi Thakur, Dhanesh Kanwar

**Affiliations:** 1 Public Health, Indian Institute of Public Health Gandhinagar, Gandhinagar, IND

**Keywords:** case finding, ntep, tb care cascade, persons with tb, patient pathway, care-seeking behavior

## Abstract

Background: Tuberculosis (TB) continues to pose a significant public health challenge globally. Despite efforts to meet targets set by the End-TB Strategy, progress has been slow. Health-seeking practices that decide approaches to various sectors of healthcare providers result in inappropriate diagnosis and lack of awareness regarding available standard treatment, indicating inaccuracy in estimated incidences and underreporting.

Objective: This study was designed to map the patient pathways for Persons with Tuberculosis (PwTB) from their initial point of contact through to diagnosis and treatment. It aimed to identify the socio-demographic characteristics and profiles of PwTB, as well as their choice of healthcare facilities, that influenced care-seeking behavior throughout the TB care cascade.

Methods: A cross-sectional study was conducted from January to July 2022 in Jharkhand and Gujarat, India. Data were collected from 997 PwTB using a pre-designed structured questionnaire, covering socio-demographic profiles, TB profile of PwTB, and care-seeking behavior. The study analyzed the number and types of facilities visited, categorized the data, and used chi-square and binary logistic regression tests to identify significant associations.

Results: In a study of 965 TB patients, 58.8% were male, and 61.3% were aged 18-40. Patients visited an average of two healthcare facilities, with significant associations found between age, occupation, comorbidity status, and facility switching (p < 0.005). Public health facilities were the primary point of care, with 91.4% using them for first consultations and 80.6% for treatment. Regression analysis highlighted significant predictors of care-seeking behavior, underscoring the need to enhance public healthcare infrastructure.

Conclusion: Understanding patient pathways and the factors influencing care-seeking behavior is crucial for improving TB management. Strengthening public healthcare infrastructure and enhancing coordination between public and private sectors can reduce transitions and ensure timely and appropriate care.

## Introduction

Tuberculosis (TB), although treatable, is one of the leading causes of death from infectious diseases worldwide. Efforts to achieve the targets set by the End-TB Strategy have been progressing slowly [1]. In 2022, 7.5 million people were newly diagnosed with TB and officially notified as TB cases [2]. However, there are still significant gaps between the estimated number of people who develop TB each year and those who are officially reported. These gaps can be attributed to several factors, including limited access to healthcare services in remote or underserved areas, where diagnostic facilities may be scarce or difficult to reach. Additionally, socio-economic barriers such as poverty, stigma, and lack of awareness can deter individuals from seeking timely medical attention. Moreover, in some regions, healthcare systems may lack the necessary infrastructure, trained personnel, or resources to accurately diagnose and report TB cases. This suggests challenges in care-seeking behavior at the initial stages of infection [3].

India, in particular, faces a substantial TB burden. In 2022, India recorded a high notification of 24.2 lakh TB cases, an increase of over 13% compared to 2021, with a treatment initiation rate of 95.5% among the notified cases. The National TB Prevalence Survey 2019-21 found that up to 64% of individuals with presumptive TB symptoms in the general population did not seek care, despite the National Tuberculosis Elimination Programme (NTEP) providing free and quality anti-TB drugs for patients in both public and private sectors [1-4].

A study in Indonesia, which has one of the highest proportions of non-notified TB cases globally, revealed that an accurate and widely available diagnostic test for TB could significantly reduce delays, transmission, and mortality [3]. Similar issues are observed in India, where patient pathways to TB care often involve various treatment-seeking behaviors and delays, particularly when patients initially visit private healthcare facilities [5].

Delays in diagnosis and treatment initiation have a profound impact on TB treatment outcomes. These delays can lead to prolonged infectious periods, increased transmission, and higher mortality rates [6]. The process typically involves symptom recognition, understanding the illness, initial home care and monitoring, and eventually seeking care at a health facility, followed by medication adherence [7]. The quality of care and treatment adherence, especially from private practitioners, can vary widely, highlighting the need for robust public-private collaborations to ensure consistent and effective TB management [8,9].

To address the persistent gaps in TB care, in the era of prompt diagnosis and treatment initiation, it is necessary to map patient pathways and identify the factors influencing care-seeking behavior [10,11]. By pinpointing these transitions of facilities in TB care, targeted interventions can be developed to streamline the TB care cascade, decrease underreporting, and ultimately enhance treatment outcomes in high-burden regions like Jharkhand and Gujarat [12,13].

Improving TB care and management is crucial for bridging the existing gaps, ultimately contributing to the larger goal of TB eradication. By understanding the factors that influence care-seeking behavior, the research will provide valuable insights for improving TB care and management.

## Materials and methods

Study design

A cross-sectional study was conducted from January to July 2022 in two Indian states, Jharkhand and Gujarat. The data in this study was collected by interviewing Persons with Tuberculosis (PwTB) using a predesigned data collection tool.

Study setting

The assessment was undertaken in 10 out of 25 tuberculosis units (TUs) in the Purbi Singhbhum and Ranchi districts of Jharkhand and 22 out of 32 TUs in the Gandhinagar and Surat districts of Gujarat. These states and districts were selected in consultation with state officials to ensure a representative sample from diverse geographies having varied performance in NTEP.

Sampling and data collection

The data for this study were part of a larger operational research project examining reasons for delays in the TB care cascade [14]. A quantitative assessment was conducted using a pre-defined, structured questionnaire, allowing a comprehensive understanding of the patient pathway including sections on socio-demographic profiles, clinical profiles, and care-seeking behavior of PwTB, such as the types and frequency of facilities visited at different stages of the TB care cascade.

In the present study, the TU of the block served as the sampling unit from the selected districts. The probability proportional to size (PPS) sampling method was used to select these units, considering patient load and the distribution of operational TUs. In total, 32 out of 57 TUs were randomly selected based on the PPS principle. A multistage cluster sampling was used to recruit the patients. From each TU, patients were recruited randomly depending on their availability and willingness to participate. The assessment was intended to cover all stages of the TB care cascade and the facilities visited by study subjects in the respective phases. Notified TB patients were recruited from the 1st Quarter 2019 to the 4th Quarter 2020 for DS-TB. Similarly, for drug-resistant TB (DRTB), patients were recruited from the 3rd Quarter 2018 to the 4th Quarter 2020. Patients were interviewed at their convenience, either at the hospital or at their residences.

Based on the formative research experiences in the selected study sites, it has been averaged out that 50% of the PwTB visited more than two healthcare facilities during their care-seeking pathway. Therefore, with 95% confidence interval (CI), 90% power, and 50% facility switching, the estimated sample size for the one-sample proportion test (Wald Z test) was 785. Adding a non-response rate of an average of 10%, the final sample size for the study was 872. Hence, 997 respondents were interviewed, with 594 participants from Gujarat and 403 from Jharkhand. After an extensive data cleaning process to ensure accuracy and reliability, the final sample size was adjusted to 965 respondents, comprising 575 from Gujarat and 390 from Jharkhand. This cleaning process involved removing incomplete, inconsistent, or problematic data entries, enhancing the validity of the study's findings.

Inclusion criteria

Inclusion criteria were (i) individuals who had been diagnosed with TB and officially registered on the Ni-kshay Portal within the specified study regions (Purbi Singhbhum and Ranchi districts of Jharkhand, and Gandhinagar and Surat districts of Gujarat) during the study period (January to July 2022) and (ii) individuals who had completed their TB treatment within the study duration.

Exclusion criteria

Exclusion criteria were (i) individuals who did not agree to participate in the study or refused to provide informed consent, (ii) individuals who were severely ill and not available for interaction or assessment at the time of the interview visits, and (iii) individuals who were transferred out from the selected TUs and districts at the time of the visits.

Ethical consideration

The study received approval from the Institutional Ethics Committee of the Indian Institute of Public Health Gandhinagar. Informed consent was obtained from all participants after explaining the study's purpose, procedures, risks, and benefits. Confidentiality was maintained by anonymizing data and securely storing it.

Data analysis

To understand care-seeking behavior, samples were categorized based on the average number of facilities visited by the PwTB eligible for the study to seek healthcare services at a designated healthcare establishment. The categories were: (i) study subject visited two or fewer facilities (≤ 2) and (ii) study subject visited more than two facilities (> 2)

Data was entered into an Excel file (MS Excel 2021, Microsoft Corporation, Redmond) and imported into IBM SPSS Statistics for Windows, Version 25 (Released 2017; IBM Corp., Armonk, New York, United States) for analysis. The patient data on various variables have been summarized using numbers and proportions. The chi-square test was used to compare groups, while the test examined linear trends. Risk measures were determined using adjusted odds ratios (aORs) and 95% CI, with the level of significance set as p-value <0.05. Two key parameters were quantified as: (a) study subject visited two or fewer facilities and (b) study subject visited more than two facilities. The primary outcome variable was descriptive; the dichotomous variables were compared with the input variables. The input variables were socio-demographic variables, clinical profile variables, and variables related to care care-seeking behavior of PwTB. 

Logistic regression was used to identify variables independently associated with the facility visits by the study population. Binary logistic regression was done by the ‘enter’ method, considering more than two facilities visited by the study population as the dependent variable and entering the following variables as the independent variables in Step 1: age in years, occupation, type of case, site of disease, comorbidity, first point of contact, first formal consultation, diagnostic facility, and treatment facility. Bivariate analysis and logistic regression were reported as chi-square test and aORs, respectively, with 95% CIs.

## Results

This study enrolled 965 individuals diagnosed with TB, providing valuable insights into their demographic characteristics and care-seeking behaviors across two Indian states, Gujarat and Jharkhand. The study revealed a wide-ranging pattern in healthcare facility utilization among PwTB. In Gujarat, PwTB accessed between one and six facilities, whereas in Jharkhand, this range extended from one to fourteen facilities, with a mean of two facilities visited overall. This variability underscores the complex care-seeking behaviors influenced by geographic, socioeconomic, and health system factors.

Characteristics of the study population

The study observed that around 20.31% (n=196) of PwTB visited more than two healthcare facilities in their care-seeking pathway. Out of all PwTB (N = 965), 61.3% (n=592) of the PwTB belong to 18 - 40 years. Only 6.2 % (n=60) were more than 60 years and above. The distribution of more than two health facilities (n = 196) visits among the age groups was 68.8% (n=135) for 18 - 40 years, followed by 14.2%, 11.7%, and 5.1 % in respective 41-60 years, ≤ 17 years, and > 60 years. The association of categories with outcome was found statistically significant, (p< 0.005), with the young adult population having 1.71 (95 %CI- 0.790-3.697) times higher odds of having more than two health facility visits. Similarly, occupation was also found to be statistically significant (p< 0.005) with students (29.1%) and housewives (22.4%) having more proportion of health facility visits compared to others (Table 1).

**Table 1:** Profile of PwTB based on care-seeking behavior. * Socio-economic status was classified as per B.G. Prasad Classification 2024 [15]. ** indicates statistically significant variables PwTB: Persons with Tuberculosis

Variables	Total no. of facilities visited			Adjusted odd ratio (aOR)	95% CI	Chi-square test (p-value)
	Less than or equal to two facilities	More than two health facilities	Total N (%)			
Age**						
≤17 years	63 (73.2%)	23 (26.7%)	86 (8.9%)	1.563	0.591-4.135	χ² = 13.887 (<0.005)
18-40 years	457 (77.2%)	135 (22.8%)	592 (61.3%)	1.709	0.790-3.697	
41-60 Years	199 (87.6%)	28 (12.4%)	227 (23.52%)	0.968	0.416-2.251	
>60 Years	50 (83.3%)	10 (16.7%)	60 (6.2%)	Ref.		
Gender						
Male	457 (80.6%)	110 (19.4%)	567 (58.7%)	1.431	0.903-2.269	χ² = 0.704 (0.401)
Female	312 (78.4%)	86 (21.6%)	398 (41.3%)	Ref.		
Education						
Literate	624 (78.7%)	169 (21.3%)	793 (82.2%)	1.004	0.580-1.737	χ² = 2.752 (0.097)
Illiterate	145 (84.3%)	27 (15.7%)	172 (17.8%)	Ref.		
Occupation**						
Student	95 (70.9%)	39 (29.1%)	134 (13.8%)	Ref.		χ² = 17.856 (<0.005)
Employed	124 (84.4%)	23 (15.6%)	147 (15.3%)	0.824	0.413-1.644	
Housewives	149 (77.6%)	43 (22.4%)	192 (19.8%)	1.039	0.563-1.918	
Daily wage workers	176 (87.2%)	26 (12.8%)	202 (20.9%)	0.839	0.431-1.634	
Self-employed	121 (80.1%)	30 (19.9%)	151 (15.7%)	1.028	0.527-2.005	
Other	104 (74.8%)	35 (25.2%)	139 (14.5%)	1.377	0.736-2.577	
Socio-economic status*						
Class 1	40 (80.0%)	10 (20.0%)	50 (5.2%)	1.137	0.464-2.787	χ² = 1.782 (0.776)
Class 2	98 (76.6%)	31 (23.4%)	129 (13.4%)	1.378	0.751-2.526	
Class 3	165 (80.8%)	39 (19.2%)	204 (21.1%)	1.079	0.624-1.867	
Class 4	253 (79.1%)	67 (20.9%)	320 (33.2%)	1.215	0.767-1.925	
Class 5	213 (81.3%)	49 (18.7%)	262 (27.1%)	Ref.		
Type of case**						
New	625 (80.8%)	148 (19.2%)	773 (80.10%)	0.529	0.287-0.975	χ² = 12.172 (<0.005)
Retreatment	106 (80.3%)	25 (19.7%)	131 (13.6%)	0.519	0.243-1.106	
PMDT	38 (62.3%)	23 (37.7%)	61 (6.3%)	Ref.	-	
Site of disease**						
Pulmonary	596 (78.2 %)	166 (21.8%)	762 (79%)	1.762	1.111-2.794	χ² = 4.862 (<0.005)
Extrapulmonary	173 (85.2 %)	30 (14.8%)	203 (21%)	Ref.	-	
Comorbidity**						
No	161 (98.2%)	3 (1.8%)	164 (17%)	0.073	0.023-0.236	χ² = 41.696 (<0.005)
Yes	608 (75.9%)	193 (24.1%)	801 (83%)	Ref.	-	

Among the types of TB cases, about 80% (n=773) were diagnosed as new cases. While only 6.3% of them were DRTB and others (13.6%) were previously treated for TB episodes, having a statistically significant association with health facility visits (p< 0.005). Additionally, pulmonary TB cases (21.8%) had more than two health facilities, having 1.7 (95%CI- 1.111-2.794) times higher odds of more health facility visits during the entire TB treatment. Similarly, among the 83% of those who had comorbid conditions, 24.1% were involved in visiting more than two health facilities and also showed significant association (p<0.005).

Table 2 further examined the relationship between the total number of healthcare facilities visited by PwTB and variables related to phase-wise care-seeking behavior, to find statistically significant associations.

**Table 2:** Phase-wise care-seeking practice. ** indicates statistically significant variables.

Variables	Total no. of facilities visited			Adjusted odd ratio (aOR)	95% CI	Chi-square (p-value)
	Less or equal to two facilities	More than two facilities	Total N (%)			
First point of contact**						
Informal	197 (79.8%)	50 (20.2%)	247 (25.6%)	0.689	0.454-1.046	χ² = 61.307 (<0.005)
Public health facility	329 (91.4%)	31 (8.6%)	360 (37.3%)	0.315	0.214-0.577	
Private	243 (67.8%)	115 (32.2%)	358 (37.1%)	Ref.		
First Formal Consultation**						
Public health facility	569 (87.0%)	85 (13.0%)	654 (67.6%)	0.476	0.305-0.745	χ² = 67.069 (<0.005)
Private	200 (64.3%)	111 (35.7%)	311 (32.4%)	Ref.		
Diagnostic facility**						
Public health facility	622 (84.2%)	117 (15.8%)	739 (76.6%)	0.696	0.433-1.118	χ² = 39.105 (<0.005)
Private	147 (65.0%)	79 (35.0%)	226 (23.4%)	Ref		
Treatment Facility**						
Public health facility	707 (80.6%)	170 (19.4%)	877 (91%)	1.472	0.809-2.678	χ² = 5.102 (<0.005)
Private	62 (70.4%)	26 (29.6%)	88 (9%)	Ref		

Public healthcare facility was the first point of contact for 37.3% (n=360), followed by private health facilities 37.1% (n=358). However, private (32.2%) and informal (20.2%) had higher proportions of them visiting more than two facilities to seek pre-consultation care. The association between the first point of contact and the number of facilities visited was statistically significant (p < 0.005). The first point of contact refers to the initial healthcare facility or provider approached by the patient for advice or assistance regarding symptoms, which includes informal healthcare providers like traditional healers or pharmacists, as well as formal public or private healthcare settings.

For the first formal consultation, 35.7% (n=111) visited more than two health facilities and first consulted a private facility. Compared to 13.0% (n=85), who first consulted a public health facility. This association was statistically significant (p < 0.005). The first formal consultation is defined as the initial encounter with a licensed healthcare professional at a recognized healthcare facility, where a formal assessment, diagnosis, or treatment plan is initiated.

When examining the diagnostic facility, 84.2% (n=622) visited two or fewer facilities that were diagnosed at a public health facility, while 15.8% (n=117) visited more than two. For those diagnosed at a private facility, 35.0% (n=79) visited more than two health facilities. The association between the diagnostic facility and the number of facilities visited was statistically significant (p < 0.005).

Lastly, in terms of the treatment facility, 80.6% (n=707) treated at a public health facility visited two or fewer facilities, while 19.4% (n=170) visited more than two. Among those treated at a private facility, 29.6% had more facility visits, significantly higher than those treated at a public health facility. The association was also statistically significant (p < 0.005).

Regression analysis of the predictors of facility switching

The study used a binomial logistic regression model to estimate the aOR and a 95% CI to describe the association between predictor variables and outcome variables (total no. of facilities visited). The logistic regression analysis was restricted to variables significantly associated with visiting more than two facilities, confirming notable trends. The model showed that the Nagelkerke R2 value was 0.244 with a classification accuracy of 79.7% with nine studied variables.

**Figure 1:**
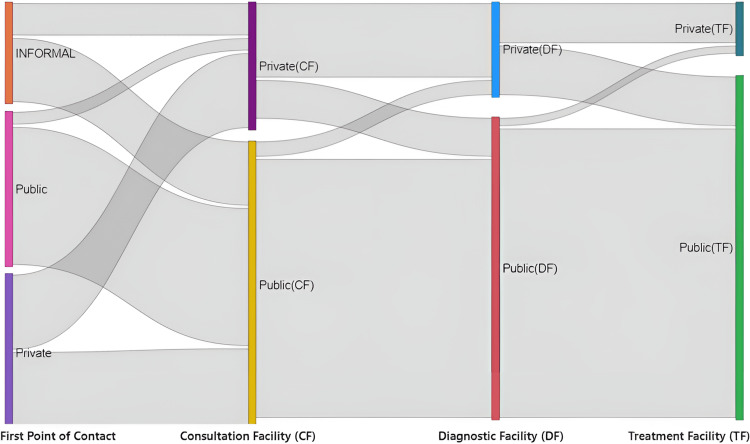
The Sankey diagram shows stage-wise dispersion of PwTB between choice of facilities (represented through different colors to indicate distinct facility from the previous stage) from the first point of contact to treatment initiation. It further describes how the cohort of PwTB (proportional to the width of grey area) flow with respect to their preferences in seeking care at different levels of the health system. PwTB: Persons with Tuberculosis

Figure 1 gives an overview that PwTB who entered the public health system as their first point of contact remained in the same type of facilities in further stages. Conversely, those preferring private and informal facilities fragmented into two proportions at the consultation stage diversifying the pathway further. Whereas, in between consultation and diagnostic phase the flow merged more towards the public type of facility, as well in the final stage of treatment initiation stage, with few transitions from public facilities to private in every stage. Overall, the graph visualizes the manifold pathways during the TB care cascade and represents how PwTB who initially began with public facilities stayed within it, representing a more streamlined care pathway, which is the most prevailing pathway amongst other pathways as this consistency spans through consultations, diagnostics, and treatment stages.

**Figure 2:**
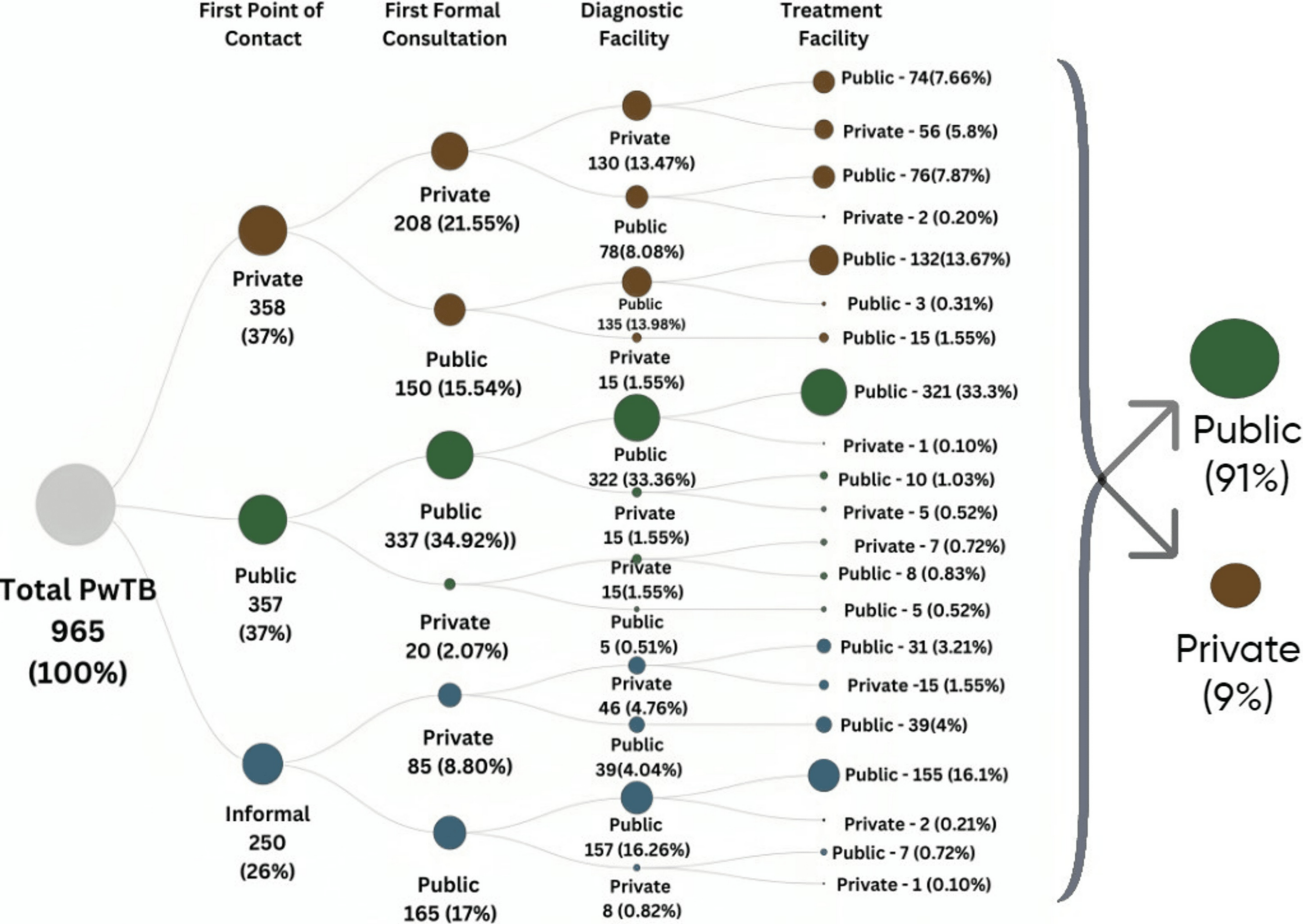
Patient pathways for tuberculosis care: flow from first contact to the treatment facility. PwTB: Persons with Tuberculosis

Figure 2 provides a more detailed visual of the proportion of the study population (represented as the size of a circle) switching between each facility within each phase. Mostly, PwTB initially visited private facilities or public health facilities both accounting for 37%, with many also seeking informal consultations, which is 26%. As the patient proceeded toward their first formal consultation only 32% ended up at a private facility, whereas the maximum consulted from the public type of facilities. In progression to their journey, three-fourth of patients availed diagnostic facility from the public sector that is 76% and only 24% claimed private diagnostic facilities. Hence, out of 358 (37%) and 250(26%) PwTB who had their first point of contact at private health facility and informal sector respectively, 300 (31%) and 232 (24%) ended up receiving treatment at a public health facility, whereas only 1.5% ended up receiving treatment at a private health facility in spite of first formal consultation at public health facility, indicating that the majority of patients inclined toward the Government facilities as the stage of infection advanced. Overall, 91% of total PwTB (N=965) end up getting treated at public health facilities, while private health facilities contribute to the remaining 9%.

## Discussion

The current TB case detection framework depicts two pathways to case detection, namely the patient-initiated pathway and a complementary provider-initiated screening pathway [16,17]. The term “patient-initiated pathway” was introduced in 2011 to improve the previously used term “passive case finding”[17]. This pathway is initiated by a person experiencing and recognizing TB symptoms. It requires people to access an appropriate health facility where a health worker may identify them as having presumptive TB and respond appropriately using a diagnostic algorithm to confirm diagnosis [18]. This study mapped patient pathways among PwTB during their entire care cascade. Findings revealed the association of different care-seeking practices and factors that might have influenced the navigation within the healthcare system for TB treatment.

The study found that 20.3% of patients changed healthcare facilities during their treatment, which aligns with findings from earlier studies in Taiwan in 2002 [19]. Another study in Taiwan in 2004 [20] reported similar findings that 34% of patients started TB treatment at a facility different from the first one they visited. These results indicate that diverse healthcare-seeking behaviors are common in different countries and can be influenced by factors such as how easy it is to access healthcare, economic situations, and other clinical factors [21].

Among the other contributing factors, the frequency of switching facilities was less among elder patients compared to young adults, which is at par with the other studies [22,23] indicating that young adults initially reached out to a pharmacy or informal healthcare provider as their first point of contact, being one of the reasons of extended episodes of TB. Previous studies, from Pakistan [24], Tanzania [25], and Bangladesh [26], highlighted various other qualitative factors contributing to these behavioral patterns, such as health system inefficiencies, poor socioeconomic status, lack of TB knowledge, and cultural beliefs.

The type of TB case, whether it is pulmonary TB or drug-resistant TB (DR-TB), significantly affects the health-seeking behavior of patients. Research indicates that individuals with pulmonary TB often visit healthcare facilities due to the greater severity of their illness, the need for additional services, and feelings of embarrassment and fear [27].In contrast, patients with DR-TB in countries like Bangladesh tend to experience delays in seeking proper healthcare, with many initially seeking care from informal providers such as drug sellers, resulting in diagnostic and treatment initiation delays [28]. It is essential for healthcare systems to understand these differences in order to develop interventions that improve knowledge, reduce delays, and enhance healthcare-seeking behavior among different types of TB cases.

PwTB who had public facilities as their first point of contact have lower odds of switching healthcare facilities compared to those having private as their first point of contact. This finding aligned with the existing literature, which suggests that public health facilities often provide more integrated and coordinated care pathways [20]. Public health facilities follow standardized protocols and offer continuity of care, reducing the need for patients to seek multiple opinions or treatments from different providers. In contrast, private healthcare providers might not always have the same level of integration, leading to fragmented care and higher instances of doctor shopping as patients seek better or alternative diagnoses and treatments [29]. Consequently, improving care coordination and accessibility within public health facilities could be an effective strategy to reduce doctor shopping practices, enhancing overall treatment adherence and health outcomes. Thus, the findings of the current study and evidence suggest that standardizing terminology, enhancing transparency, and facilitating communication among stakeholders may help in general awareness and smooth accessibility to current services [29].

Limitations

While this study offers important insights into the pathway of PwTB seeking healthcare, there are some limitations to consider. The research focused on a specific area and a small number of healthcare centers, so the findings may not apply directly to other regions with different cultural norms. Also, the study relied on patients remembering and reporting their experiences, which can sometimes lead to recall bias. Furthermore, the study utilized quantitative data to understand the healthcare utilization pattern and healthcare-seeking behavior of PwTB. To have a more comprehensive understanding, future research should investigate specific region-specific and cultural factors using a qualitative approach.

## Conclusions

The NTEP's strong case-finding approach relies on PwTB being well-informed about the need for medical care and where to access it. Patients often use both public and private diagnostic services based on availability, cost, and quality, highlighting the need for seamless integration between sectors to enhance care. Public health facilities generally see a steady patient flow, emphasizing the importance of strengthening public health infrastructure. Gender, education, and socioeconomic status do not significantly impact care experiences. Efforts such as public-private mix models, joint service delivery, integrated information systems, capacity-building programs, and financial incentives can improve outcomes by fostering collaboration between public and private sectors, enhancing TB case detection, treatment adherence, and overall patient care
